# Que/n-HA/PGCL microspheres orchestrate bone repair via context-dependent osteoimmune mechanisms: a transcriptomic dissection of direct and immune-mediated signaling

**DOI:** 10.3389/fimmu.2026.1731387

**Published:** 2026-04-30

**Authors:** Chunyu Han, Qihui Wang, Shuang Yang, Yicen Ai, Yutong Liu, Wenzhi Song, Wanzhong Yin

**Affiliations:** 1 Department of Stomatology, China-Japan Union Hospital, Jilin University, Changchun, China; 2 Department of Otolaryngology Head and Neck Surgery, The First Hospital of Jilin University, Changchun, China

**Keywords:** bone regeneration, gene, immunomodulation, RNA-seq, signaling pathway

## Abstract

**Introduction:**

Bone defect repair remains a clinical challenge due to the complex inflammatory microenvironment that regulates mesenchymal stem cell fate.

**Methods:**

Based on our previous work demonstrating that quercetin-loaded n-HA/PGCL (Que/n-HA/PGCL) nanocomposite microspheres promote bone repair via immunoregulation and osteogenesis, the present study employs RNA-seq and Western blot validation to elucidate the transcriptomic mechanisms by which these microspheres differentially regulate bone marrow mesenchymal stem cells (BMSCs) in direct versus immune-mediated microenvironments. Using a Transwell co-culture system, we simulated two distinct conditions: direct BMSC-microsphere interaction and BMSC culture with microsphere-modulated RAW264.7 macrophages.

**Results:**

RNA-seq and Western blot analyses revealed context-dependent pathway activation: direct Que/n-HA/PGCL treatment activated PI3K-Akt/MAPK signaling in BMSCs, while immune-mediated conditions activated Ras/MAPK and TGF-β/Smad pathways via macrophage modulation.

**Discussion and conclusion:**

By identifying the context-dependent activation of PI3K-Akt/MAPK pathways in direct BMSC interaction and Ras/MAPK/TGF-β/Smad pathways in immune-mediated scenarios, this work offers a mechanistic blueprint for the development of intelligent bone repair materials. The success of bone defect repair is intricately linked to the complex inflammatory microenvironment, which dictates the fate of mesenchymal stem cells.

## 1 Introduction

In our previous studies (1), we developed a composite microsphere composed of quercetin (Que), nano-hydroxyapatite (n-HA), and poly(glycolide-co-epsilon-caprolactone) (PGCL). The 4 wt% Que/n-HA/PGCL formulation was identified as optimal based on thorough characterization of physicochemical properties and drug release kinetics. Using immunofluorescence, flow cytometry, ALP staining, and Alizarin Red S, we demonstrated that quercetin released from these microspheres not only directly promoted BMSC osteogenic differentiation but also indirectly modulated osteoblast behavior by promoting M2 macrophage polarization.

Quercetin, a natural flavonoid with potent antioxidant and anti-inflammatory properties, has been extensively studied for its osteogenic potential (2). Previous studies have demonstrated that quercetin promotes osteogenic differentiation of BMSCs through multiple signaling pathways, including MAPK (3), PI3K-Akt (2), and BMP (4). Additionally, quercetin exerts immunomodulatory effects by promoting M2 macrophage polarization and suppressing pro-inflammatory cytokine production, thereby creating a favorable microenvironment for bone regeneration (5).

Bone regeneration is orchestrated by intricate interactions between immune cells and skeletal stem cells (6). Macrophages, as key regulators of the inflammatory microenvironment, undergo phenotypic polarization from pro-inflammatory M1 to pro-regenerative M2 subtypes, which secrete cytokines that modulate mesenchymal stem cell osteogenic differentiation (7). Recent studies have demonstrated that immunomodulatory biomaterials can promote bone regeneration by inducing macrophage M2 polarization through various mechanisms, including metabolic reprogramming and piezoelectric stimulation (8). Emerging evidence highlights the critical role of this osteoimmune interplay in bone repair, with several signaling pathways—including PI3K-Akt, MAPK, and TGF-β/Smad—mediating macrophage-osteoblast crosstalk (9, 10). For example, hierarchically structured nanofibrous scaffolds have been shown to spatiotemporally mediate the osteoimmune microenvironment and promote osteogenesis in inflammatory conditions such as periodontitis-related alveolar bone defects (11).

However, a critical knowledge gap remains. While previous studies including our own (1) have established the phenotypic effects of quercetin-based materials on BMSCs and macrophages (enhanced osteogenic differentiation and M2 polarization), the underlying molecular mechanisms by which these materials differentially regulate osteogenesis in distinct microenvironmental contexts remain largely unexplored. Specifically, it is unknown whether Que/n-HA/PGCL microspheres activate the same or different signaling pathways when they act directly on BMSCs versus when they act indirectly through macrophage modulation. This distinction is crucial for understanding how a single biomaterial can orchestrate the complex immune-osteogenic crosstalk that drives successful bone regeneration. To systematically investigate these molecular mechanisms, we employed RNA sequencing (RNA-seq)—a hypothesis-generating tool that enables genome-wide profiling of transcriptional changes (12, 13).

Building upon our previous work, the present study aims to further investigate the osteogenic mechanisms of Que/n-HA/PGCL nanocomposite microspheres in immune-mediated versus non-immune-mediated microenvironments. The 4 wt% Que/n-HA/PGCL nanocomposite microspheres, previously identified as the optimal formulation, were used as the experimental group, while n-HA/PGCL nanocomposite microspheres served as the control group. A Transwell co-culture system was employed to simulate the early inflammatory microenvironment, with RAW264.7 macrophages and microspheres co-cultured in the lower chamber, and BMSCs seeded in the upper chamber of the Transwell insert. To mimic the later-stage osteogenic environment, BMSCs were cultured alone with the microspheres. RNA-seq was performed to compare the differentially expressed genes (DEGs) between the two groups under the two culture conditions. Subsequently, Gene Ontology (GO) and Kyoto Encyclopedia of Genes and Genomes (KEGG) pathway enrichment analyses were conducted to identify the most significant transcriptional changes between the groups. This approach allowed us to uncover the key genes and associated signaling pathways through which Que/n-HA/PGCL nanocomposite microspheres synergistically regulate osteogenesis, thereby providing a more robust theoretical basis for their enhanced application in bone tissue repair. Collectively, while the osteoimmunomodulatory potential of quercetin-based materials is recognized, the precise and compartmentalized molecular mechanisms by which they differentially regulate mesenchymal stem/stromal cells (BMSCs) in distinct microenvironmental contexts remain a significant knowledge gap. Previous research, including our own, has primarily focused on establishing the therapeutic efficacy and associated cellular phenotypes, such as macrophage polarization. The present study addresses this gap by providing the first transcriptomic dissection that decouples direct material-cell interactions from immune cell-mediated paracrine effects. By employing a Transwell co-culture system coupled with RNA sequencing, we uniquely map the divergent signaling landscapes in BMSCs: identifying the PI3K-Akt and MAPK pathways as central to direct osteoinduction, and the Ras/MAPK and TGF-β/Smad pathways as critical for immune-mediated osteogenesis. This work shifts the understanding from a phenomenological observation of enhanced bone repair to a mechanistic blueprint of context-dependent signal transduction, offering novel insights for the rational design of intelligent biomaterials that can sequentially modulate immune responses and direct osteogenic differentiation.

## 2 Methods

### 2.1 Cell culture

#### 2.1.1 Bone marrow-derived mesenchymal stem/stromal cells culture

BMSCs were purchased from Procell Biotechnology Co., Ltd. (Wuhan, China). The cells were isolated from the bone marrow of Sprague-Dawley rats and characterized by the provider for standard mesenchymal markers (CD90+, CD29+, CD45-). Culture medium was replaced after 48 h and every 2 days. At 80–90% confluency (day 4), cells were washed with PBS, treated with 0.25% trypsin for 2 min (37 °C), and neutralized with fresh medium. After centrifugation (1000 rpm, 5 min), cells were resuspended and passaged at 1:2 ratio, with medium changes every 2 days.

#### 2.1.2 RAW264.7 cells culture

The RAW264.7 macrophages were purchased from Suzhou Haixing Biotechnology Co., Ltd. The RAW264.7 macrophages were rapidly transferred to a 37 °C water bath for resuscitation. After addition of 10 mL high-glucose DMEM medium (containing 10% FBS and 1% penicillin/streptomycin), cells were centrifuged at 1000 rpm for 5 min, supernatant was discarded, and pellets were resuspended in fresh medium before seeding into 10 cm culture dishes. Cells were cultured in a 37 °C, 5% CO_2_ humidified incubator. Upon reaching 90% confluency, the old medium was aspirated, and cells were gently washed once with sterile PBS. Subsequently, 5 mL PBS were added, and adherent cells were detached using a sterile cell scraper. The cell clumps were dispersed by pipetting, transferred to a 10 mL centrifuge tube, and centrifuged at 1000 rpm for 5 min. After discarding the supernatant, cells were resuspended in fresh medium and passaged at a 1:3 ratio into new 10 cm dishes, followed by incubation at 37 °C, 5% CO_2_, and saturated humidity.

### 2.2 Que/n-HA/PGCL and n-HA/PGCL microsphere fabrication

For the control group, 1 g of PGCL and 0.25 g of n-HA were dissolved and dispersed in 7 mL of N-methyl-2-pyrrolidone (NMP). For the experimental group, quercetin (Que), accounting for 4 wt% of the total feedstock, was added the n-HA/PGCL suspension. The prepared suspension was ultrasonicated for 30 min and stirred overnight in the dark to ensure uniform dispersion. The well-dispersed suspension was then processed into microspheres using an air-flow shearing method, as previously reported (1). Specifically, the suspension was loaded into a 2 mL syringe, and the extrusion rate, controlled by a syringe pump (Longer Pump, LSP01-1A), was set to a plunger advancement speed of 0.5 cm/min, corresponding to a volume flow rate of approximately 17 mL/h for the 2 mL syringe used. The air-flow rate was maintained at 10 L/min, with the liquid surface height set at 7 cm. A 27G needle was used, and the droplets formed at the needle tip were subjected to shearing forces from the air-flow in a three-way junction, causing premature detachment and deposition into the receiving medium (water: ethanol = 6:4). The NMP in the droplets was exchanged with water, leading to solidification and microsphere formation.

### 2.3 Transwell co-culture

To model the immune-mediated paracrine effects, RAW264.7 macrophages (2 × 10^5^ cells/well) were seeded together with the microspheres in the lower chamber of a 6-well plate. Simultaneously, BMSCs (5 × 10^4^ cells/well) were seeded into the upper chamber of the Transwell insert (pore size 0.4 μm). This setup was maintained for 7 days, allowing macrophage-derived soluble factors to diffuse and act on the BMSCs.

### 2.4 RNA extraction

**Figure 1:**
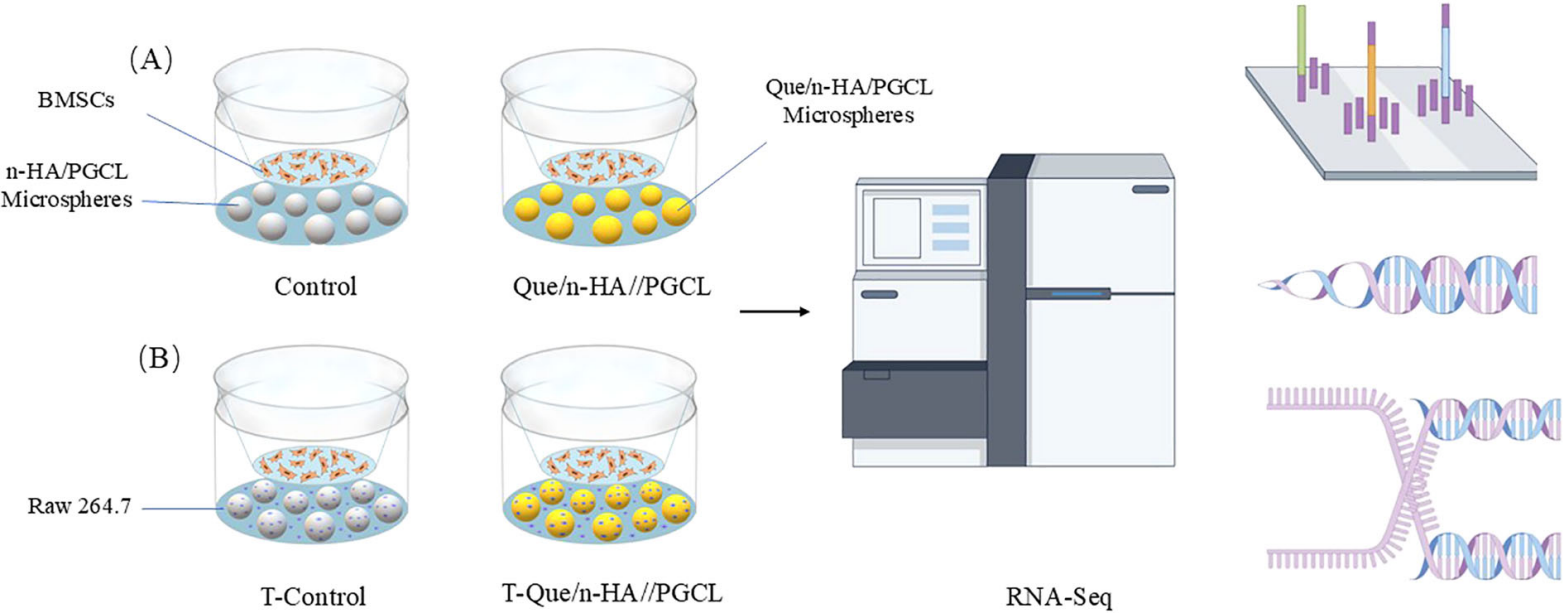
Schematic of the experimental design. **(A)** Direct culture: BMSCs cultured alone with n-HA/PGCL (Control) or Que/n-HA/PGCL (Que) microspheres. **(B)** Transwell co-culture: BMSCs were seeded in the upper chamber, while RAW264.7 macrophages and microspheres were co-cultured in the lower chamber (T-Control and T-Que groups). After 7 days, RNA was extracted exclusively from the BMSCs for sequencing.

Based on the results of preliminary experiments, the 4 wt% Que/n-HA/PGCL group was selected as the experimental group, while the n-HA/PGCL group served as the control. Bone marrow-derived mesenchymal stem/stromal cells (BMSCs) and macrophages (RAW264.7) were used for this study. As shown in **Figure 1**, direct culture was that BMSCs cultured alone with n-HA/PGCL (Control) or Que/n-HA/PGCL (Que) microspheres, Transwell co-culture was that BMSCs were seeded in the upper chamber, while RAW264.7 macrophages and microspheres were co-cultured in the lower chamber (T-Control and T-Que groups). The first two groups (Control and Que) examined the direct regulatory effects of Que/n-HA/PGCL microspheres on BMSCs, while the latter two groups (T-control and T-Que) assessed the indirect regulatory effects mediated by the immune microenvironment. BMSCs (5 × 10^4^ cells/well) and RAW264.7 (5 × 10^4^ cells/well) were seeded in six-well plates and the upper chamber of Transwell inserts, respectively, with three replicates per group. After 7 days of co-culture, BMSCs in the upper chamber were collected, only the BMSCs in the upper chamber were collected for total RNA extraction using TRIzol reagent. The RNA samples were transferred to RNase-free tubes and sent to BGI Genomics for sequencing.

### 2.5 RNA-seq data analysis

#### 2.5.1 RNA extraction and quality control

Total RNA was extracted from BMSCs using TRIzol reagent (Invitrogen, Carlsbad, CA, USA) following the manufacturer’s protocol. RNA purity was assessed using a NanoDrop 2000 spectrophotometer (Thermo Fisher Scientific, Waltham, MA, USA), and RNA integrity was evaluated using an Agilent 2100 Bioanalyzer (Agilent Technologies, Santa Clara, CA, USA). Only samples with RNA integrity number (RIN) ≥ 7.0 were used for subsequent library preparation.

#### 2.5.2 Library preparation and sequencing

mRNA was enriched from 1 μg of total RNA using oligo(dT) magnetic beads (polyA selection). The enriched mRNA was fragmented into short fragments (200–300 bp) using fragmentation buffer. First-strand cDNA was synthesized using random hexamer primers and reverse transcriptase, followed by second-strand cDNA synthesis. The double-stranded cDNA was subjected to end repair, A-tailing, and adapter ligation. The ligated products were amplified by PCR to generate the final cDNA library. Library quality and concentration were assessed using an Agilent 2100 Bioanalyzer and Qubit 2.0 fluorometer (Thermo Fisher Scientific), respectively.

The qualified libraries were sequenced on an Illumina NovaSeq 6000 platform (BGI Genomics, Shenzhen, China) with paired-end 150 bp read length, generating approximately 20 million reads per sample.

#### 2.5.3 Read alignment and quantification

Raw sequencing reads were filtered using Trimmomatic (v0.39) to remove adapter sequences, low-quality bases (Q < 20), and reads shorter than 50 bp. Clean reads were aligned to the Rattus norvegicus reference genome (Rnor_6.0, Ensembl v96) using HISAT2 (v2.1.0) with default parameters. Aligned reads were sorted and indexed using SAMtools (v1.9). Gene expression levels were quantified using featureCounts (v2.0.0), with reads counted at the gene level based on Ensembl v96 annotation. Normalized expression values were calculated as transcripts per million (TPM) for visualization.

#### 2.5.4 Differential expression and functional enrichment analysis

Differential expression analysis was performed using DESeq2 (v1.30.0) in R (v4.0.3). Genes with |log2 fold change (FC)| ≥ 1 and adjusted P-value (Padj) < 0.05 (Benjamini-Hochberg false discovery rate correction) were considered significantly differentially expressed genes (DEGs).

Gene Ontology (GO) enrichment analysis and Kyoto Encyclopedia of Genes and Genomes (KEGG) pathway enrichment analysis were performed using the clusterProfiler (v4.0.0) R package. Enriched terms were identified using the hypergeometric test, with Padj < 0.05 considered statistically significant. The reference gene set for enrichment analysis was the background of all expressed genes in the corresponding comparison.

### 2.6 Western blot

To validate the key signaling pathways identified by RNA-seq at the protein level, Western blot analysis was performed on BMSCs from the same experimental groups described in Section 2.4. After 7 days of culture, BMSCs were harvested and lysed in RIPA buffer (Beyotime, China) containing protease and phosphatase inhibitor cocktails (Roche, Switzerland). Protein concentrations were determined using a BCA protein assay kit (Thermo Fisher Scientific, USA). Equal amounts of protein (30 μg per lane) were separated by 10% SDS-PAGE and transferred onto PVDF membranes (Millipore, USA). Membranes were blocked with 5% non-fat milk in TBST for 1 h at room temperature, then incubated overnight at 4 °C with primary antibodies against the following targets: Akt (1:1000, Cell Signaling Technology), p-Akt (Ser473; 1:1000, Cell Signaling Technology), ERK (1:1000, Cell Signaling Technology), p-ERK (Thr202/Tyr204; 1:1000, Cell Signaling Technology), Smad2 (1:1000, Cell Signaling Technology), p-Smad2 (Ser465/467; 1:1000, Cell Signaling Technology), and β-actin (1:5000, Proteintech) as a loading control. After washing with TBST, membranes were incubated with HRP-conjugated secondary antibodies (1:5000, Cell Signaling Technology) for 1 h at room temperature. Protein bands were visualized using an enhanced chemiluminescence (ECL) detection system (Tanon, China) and quantified by densitometry using ImageJ software (NIH, USA). The relative protein expression levels of p-Akt, p-ERK, and p-Smad2 were normalized to their respective total protein levels (Akt, ERK, Smad2) and expressed as fold change relative to the Control or T-Control group. All experiments were performed in triplicate, and data are presented as mean ± SD. Statistical significance was determined by one-way ANOVA followed by Tukey’s *post-hoc* test, with P < 0.05 considered significant.

### 2.7 Statistical analysis

Differential expression analysis was performed using DESeq2, with genes having |log2FC| ≥ 1 and Padj < 0.05 considered significantly differentially expressed. GO and KEGG enrichment analyses were performed using the hypergeometric test, with FDR correction for multiple comparisons. Western blot data were analyzed by one-way ANOVA followed by Tukey’s *post-hoc* test using GraphPad Prism 9.0. All data are presented as mean ± SD, with P < 0.05 considered statistically significant.

## 3 Results

### 3.1 Quality assessment of sequencing data

The quality of sequencing data was rigorously evaluated, and the key metrics are summarized in **Table 1**. For all samples, the total number of raw reads, filtered clean reads, total clean bases, and the percentages of bases with quality scores ≥20 (Q20) and ≥30 (Q30) were calculated, along with the proportion of usable clean reads. The results demonstrated that the sequencing data met high-quality standards, with Clean Reads Q20 exceeding 97% (required >90%), Clean Reads Q30 surpassing 92% (required >85%), and the Clean Reads Ratio reaching over 98% (required >95%). These findings indicate that the obtained sequencing data were of excellent quality, with minimal low-quality bases and high proportions of usable reads, thereby ensuring reliable downstream bioinformatics analysis.

**Table 1:** Statistical table for sequencing data quality.

Sample	Total raw reads (M)	Total clean reads (M)	Total clean bases (Gb)	Clean reads Q20 (%)	Clean reads Q30 (%)	Clean reads ratio (%)
Control_1	22.95	22.72	1.14	97.6	93.13	98.98
Control_2	23.92	23.65	1.18	97.49	92.84	98.85
Control_3	23.92	23.65	1.18	97.41	92.62	98.86
Que_1	23.92	23.68	1.18	97.3	92.28	99
Que_2	23.92	23.67	1.18	97.51	92.89	98.95
Que_3	23.92	23.67	1.18	97.61	93.17	98.95
T_Control_1	23.92	23.68	1.18	97.58	93.07	98.99
T_Control_2	23.92	23.68	1.18	97.64	93.24	99
T_Control_3	23.92	23.62	1.18	97.61	93.16	98.74
T_Que_1	23.92	23.67	1.18	97.66	93.3	98.92
T_Que_2	21.78	21.46	1.07	97.65	93.32	98.55
T_Que_3	23.92	23.64	1.18	97.69	93.39	98.83

The table provides detailed statistics for each sample, including total raw reads, number and percentage of clean reads after filtering, total clean bases, and the percentages of bases with Phred quality scores ≥20 (Q20) and ≥30 (Q30). All samples exceeded the required thresholds (Q20 > 97%, Q30 > 92%, Clean Reads Ratio > 98%), confirming high-quality sequencing data suitable for downstream analysis.

### 3.2 Read alignment and mapping statistics

After obtaining the clean reads, we aligned them to the reference genome sequence. As shown in **Table 2**, the alignment metrics demonstrated that all sequencing data met high-quality standards. The total mapping rate (Total Mapping) for each sample group exceeded 91%, while the proportion of uniquely mapped reads (Uniquely Mapping) surpassed 83%. These results fell within the expected range, confirming that the sequencing data from all samples in this study were of sufficient quality for reliable downstream analysis.

**Table 2:** Reference genome alignment.

Sample	Total clean reads (M)	Total mapping (%)	Uniquely mapping (%)
Control_1	22.72	91.51	85.2
Control_2	23.65	91.41	85.1
Control_3	23.65	91.6	85.47
Que_1	23.68	92	85.68
Que_2	23.67	91.75	85.14
Que_3	23.67	91.88	85.31
T_Control_1	23.68	91.55	84.66
T_Control_2	23.68	91.75	84.94
T_Control_3	23.62	91.46	83.47
T_Que_1	23.67	91.63	84.68
T_Que_2	21.46	91.69	84.71
T_Que_3	23.64	92.08	85.12

Alignment statistics of clean reads to the reference genome. The table summarizes the mapping efficiency for each sample group. Key metrics include the Total Mapping Rate (percentage of reads mapped to the genome, all >91%) and the Uniquely Mapping Rate (percentage of reads mapped to a unique genomic location, all >83%), confirming successful and specific alignment for reliable transcriptome quantification.

### 3.3 Sample correlation analysis

**Figure 2:**
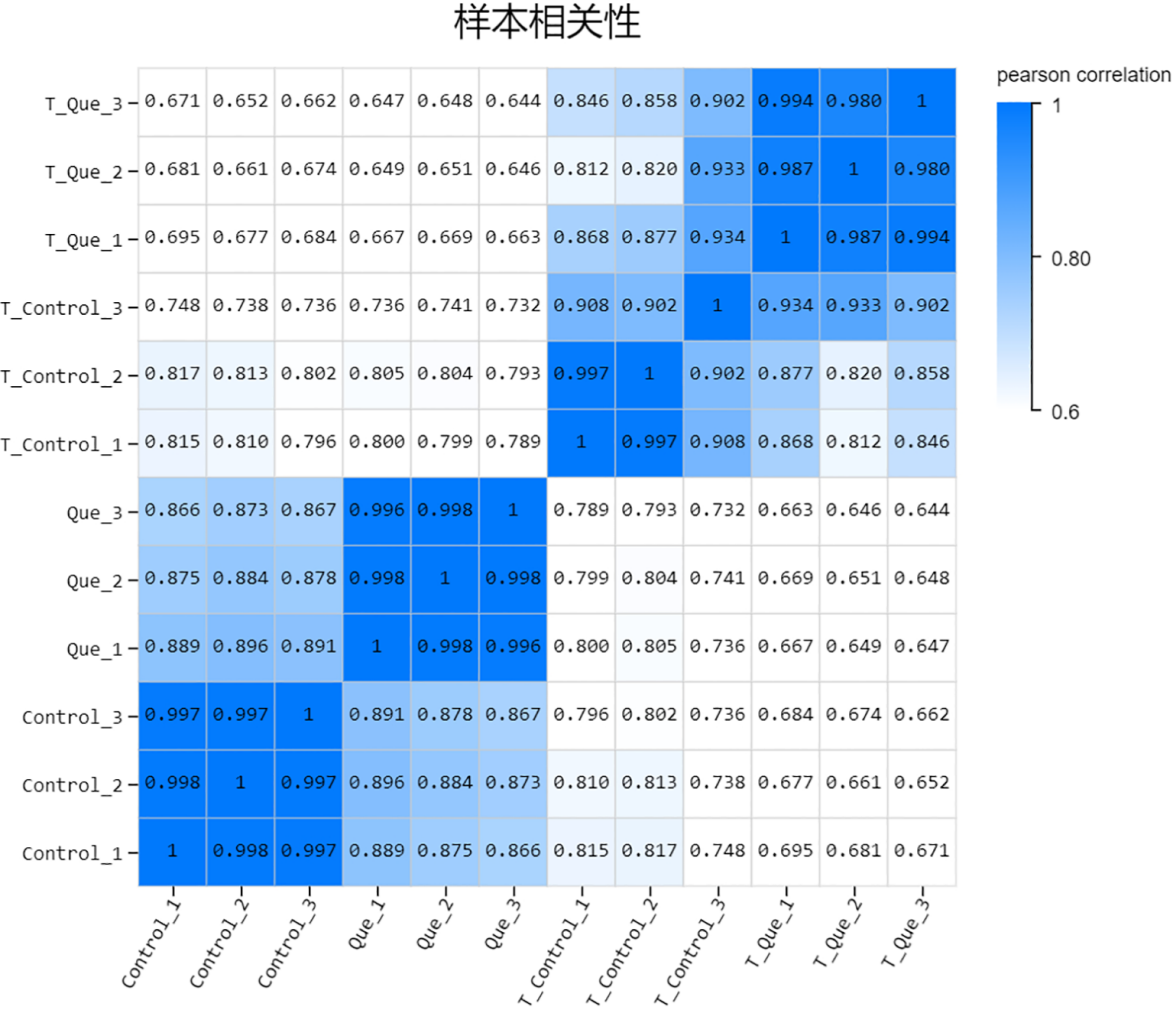
Pearson correlation analysis heatmap across all experimental samples. The matrix displays Pearson correlation coefficients (R) among all biological replicates. Darker blue indicates higher similarity, confirming strong intra-group reproducibility and distinct transcriptional profiles between experimental conditions.

To assess the similarity of gene expression patterns among samples, we calculated Pearson correlation coefficients for all pairwise comparisons of gene expression levels across the Control, Que, T-Control, and T-Que groups. The results, presented as a heatmap in **Figure 2**, revealed that all biological replicates within each group exhibited extremely high correlation coefficients (approaching 1), indicating excellent consistency in transcriptional profiles. This strong positive correlation among replicates demonstrates robust biological reproducibility and reliable data quality for subsequent differential expression analysis. Notably, the high correlation coefficients (r > 0.95) suggest minimal technical variation and consistent biological responses within each experimental group, validating the experimental design and sample preparation procedures.

**Figure 3:**
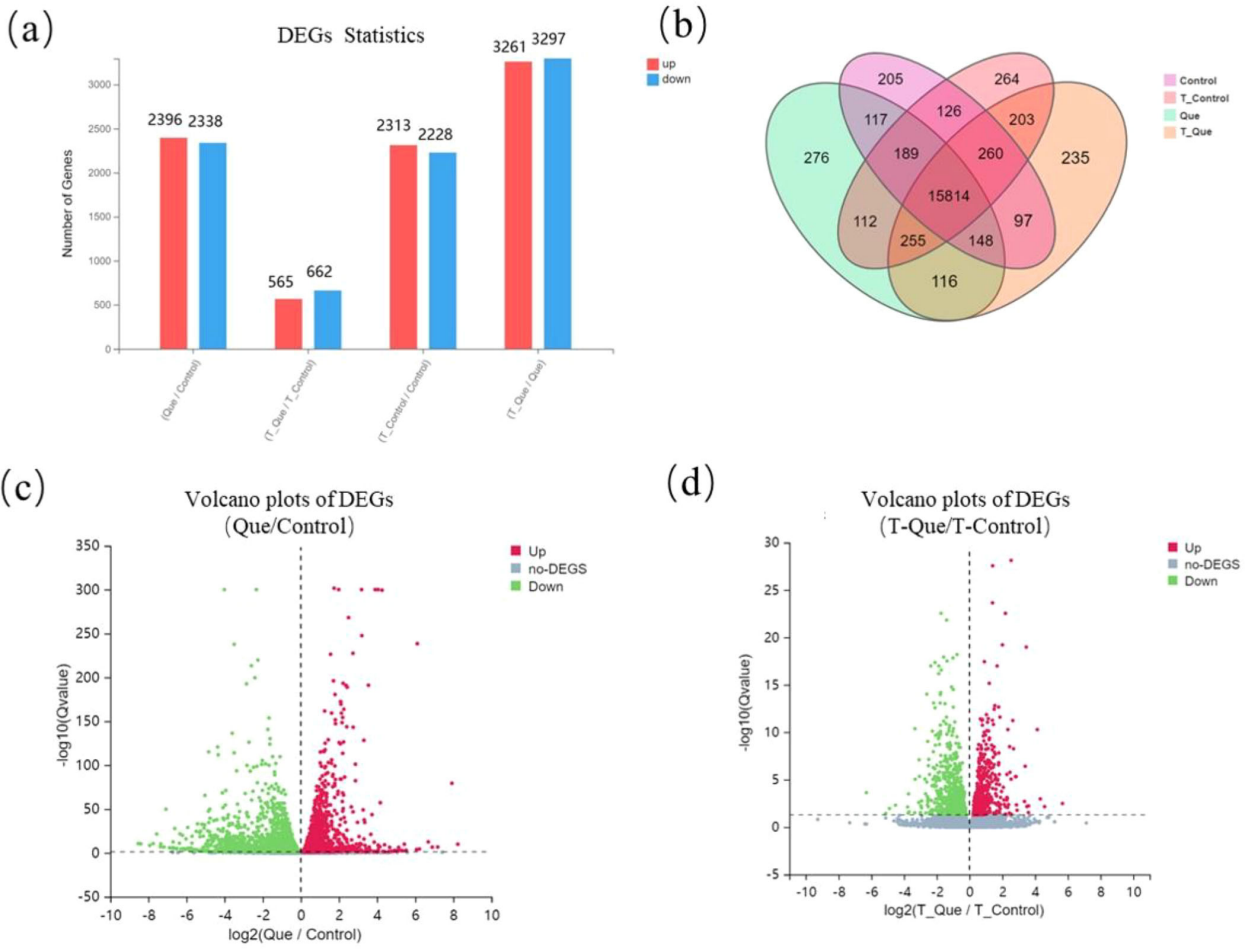
Identification of differentially expressed genes (DEGs). **(a)** Number of up/down-regulated DEGs in key comparisons. **(b)** Venn diagram illustrating overlaps of DEG sets across conditions, highlighting shared and unique responses. **(c, d)** Volcano plots visualizing the distribution of DEGs for Que vs Control **(c)** and T-Que vs T-Control **(d)**. X-axis represents log2-transformed fold change; Y-axis represents -log10-transformed Q value. Red dots: significantly upregulated genes; blue dots: significantly downregulated genes; gray dots: non-significant genes. n = 3 biological replicates per group.

Differentially expressed genes (DEGs) were identified based on a significance threshold of Q value < 0.05 and an absolute log2 fold change (FC) > 1. As shown in **Figure 3a**, compared to the Control group, the Que group exhibited 445 upregulated and 842 downregulated genes, while the T-Que group showed 159 upregulated and 273 downregulated genes relative to the T-Control group. Additionally, 711 genes were upregulated and 537 were downregulated in the T-Control group compared to the Control group, whereas 1228 genes were upregulated and 647 were downregulated in the T-Que group compared to the Que group. A Venn diagram (**Figure 3b**) was constructed to visualize the intersections of DEGs among different comparison groups, where each circle represents a unique gene set and overlapping regions indicate shared DEGs across groups. The numerical annotations in each segment denote the count of genes specific to that region or shared between groups. Furthermore, volcano plots (**Figures 3c, d**) were generated to illustrate the distribution of DEGs based on their fold change and statistical significance. The X-axis represents the log2-transformed FC values, while the Y-axis shows the -log10-transformed Q values. In these plots, red dots denote significantly upregulated DEGs, blue dots represent significantly downregulated DEGs, and gray dots indicate genes that did not meet the criteria for differential expression.

**Figure 4:**
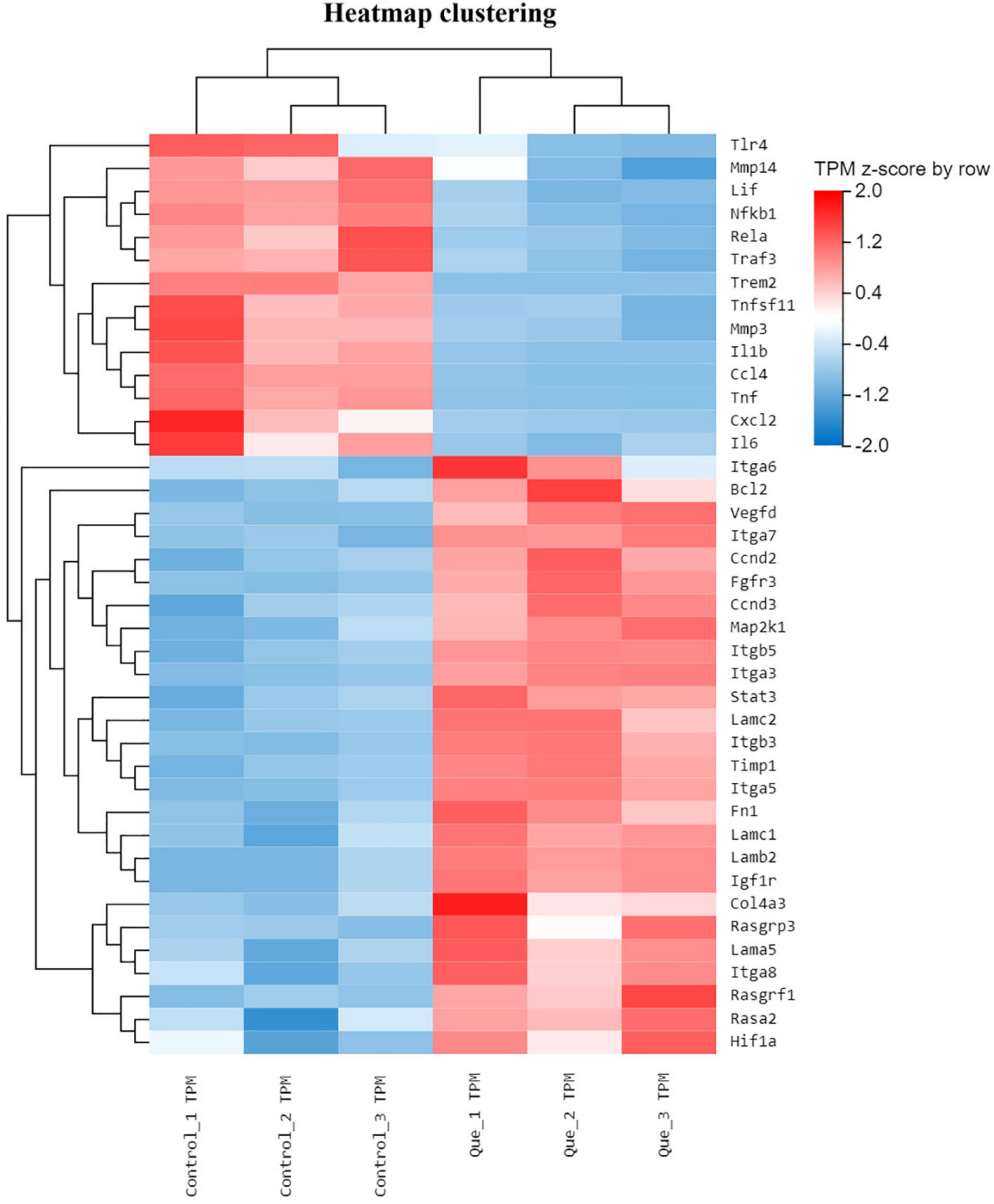
Clustered heatmap of osteogenesis-associated gene expression in BMSCs under direct culture (Que vs Control). Hierarchical clustering of 40 key DEGs associated with osteogenic differentiation. Red indicates upregulated genes; blue indicates downregulated genes. Color intensity represents the degree of differential expression (log2FC scale). Expression values are z-score normalized per gene to enable comparison across genes. Each column represents one biological replicate (n = 3 per group).

To investigate the regulatory role of Que/n-HA/PGCL nanocomposite microspheres in BMSC osteogenesis, we performed differential gene expression analysis between the Que group and Control group. From this comparison, we identified 40 differentially expressed genes (DEGs) associated with osteogenic differentiation and conducted hierarchical clustering analysis. The results, presented in **Figure 4**, demonstrate distinct gene expression patterns: blue indicates downregulated genes, while red represents upregulated genes, with deeper colors signifying more significant differential expression.

#### 3.5.1 Molecular mechanisms underlying Que/n-HA/PGCL nanocomposite microspheres-mediated osteogenic regulation in BMSCs

Compared to the Control group, RNA-seq analysis revealed that Que/n-HA/PGCL nanocomposite microspheres significantly upregulated multiple osteogenesis-related gene families in BMSCs, including the Integrin family (Itga7, Itgb5, Itga3), RAS superfamily (Rasgrp3, Rasgrf1, Rasa2), MAPK signaling pathway (Map2k1), and HIF-1α (Hif1a). Notably, Que treatment also enhanced expression of genes associated with cell adhesion (Lamc1, Lamb2, Fn1), proliferation (Ccnd2), growth signaling (Stat3, Vegfd), and collagen synthesis (Col4a3), suggesting a comprehensive activation of osteogenic pathways. Conversely, the microspheres markedly downregulated several osteogenesis-inhibitory factors, including matrix metalloproteinases (Mmp14, Mmp3), NF-κB signaling components (Nfkb1, Rela), pro-inflammatory cytokines and their receptors (IL-6, Lif, IL1β, Tnf, Tnfsf11), as well as chemokines (Ccl4, Cxcl2). This coordinated regulation of both pro-osteogenic and anti-osteogenic genes demonstrates that Que/n-HA/PGCL nanocomposite microspheres orchestrate a favorable molecular microenvironment for enhanced BMSC osteogenesis through multi-pathway modulation.

#### 3.5.2 GO enrichment analysis of differentially expressed genes

Gene Ontology (GO) enrichment analysis was performed to systematically investigate the biological functions of differentially expressed genes (DEGs) between the Que and Control groups, which was categorized into three main functional domains: molecular function, cellular component, and biological process. In the bar charts, yellow dots indicate the number of DEGs annotated to each GO term, while the length of the blue bars represents the enrichment significance (Q value), with longer bars indicating greater statistical significance.

**Figure 5:**
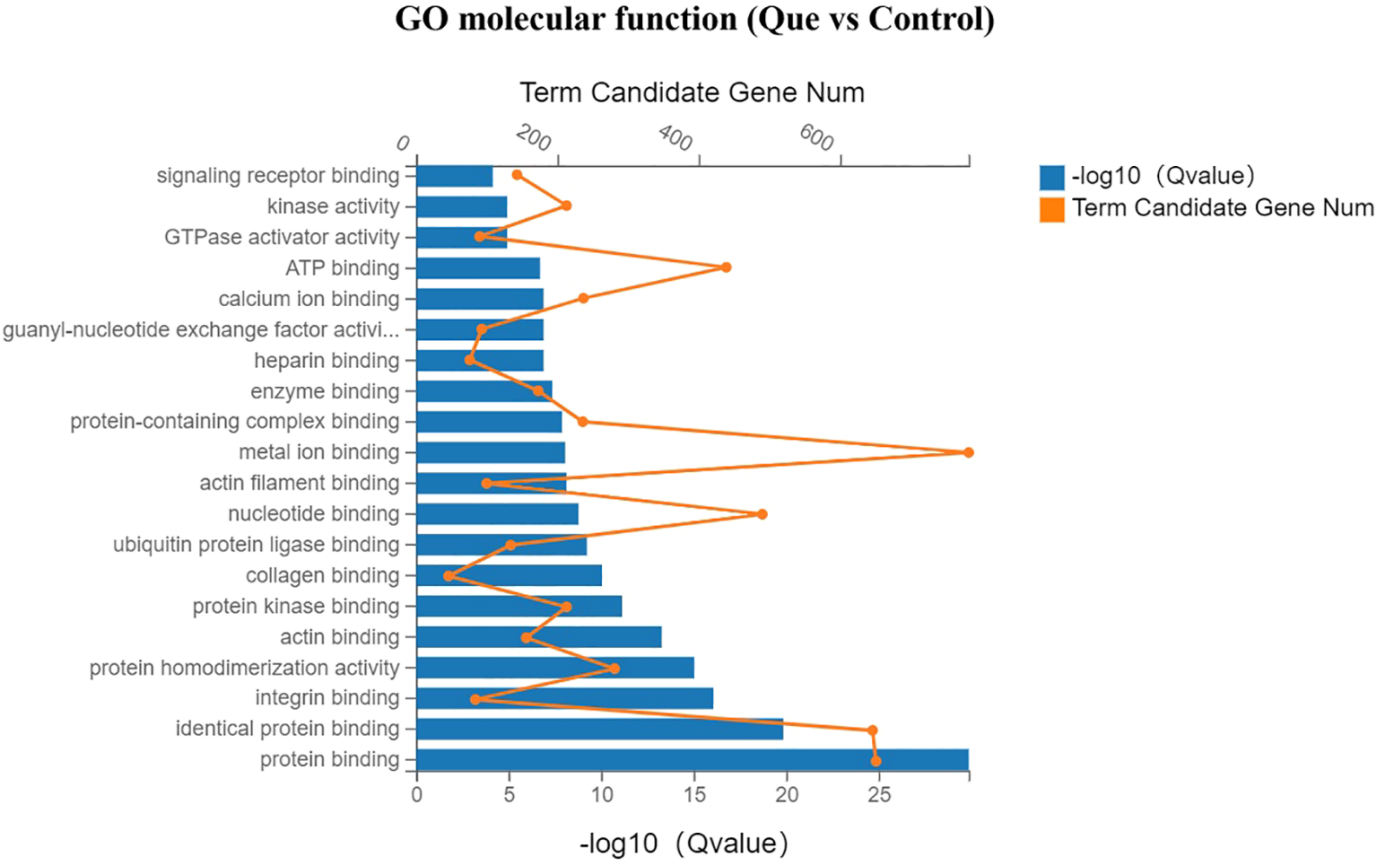
GO enrichment analysis (Molecular Function) for DEGs under direct culture (Que vs Control). Bar chart showing the top enriched GO terms. Yellow dots indicate the number of DEGs annotated to each GO term. Blue bars represent the enrichment significance (-log10 Q value), with longer bars indicating greater statistical significance. Enrichment analysis was performed using the hypergeometric test with FDR correction (Q < 0.05 considered significant).

**Figure 6:**
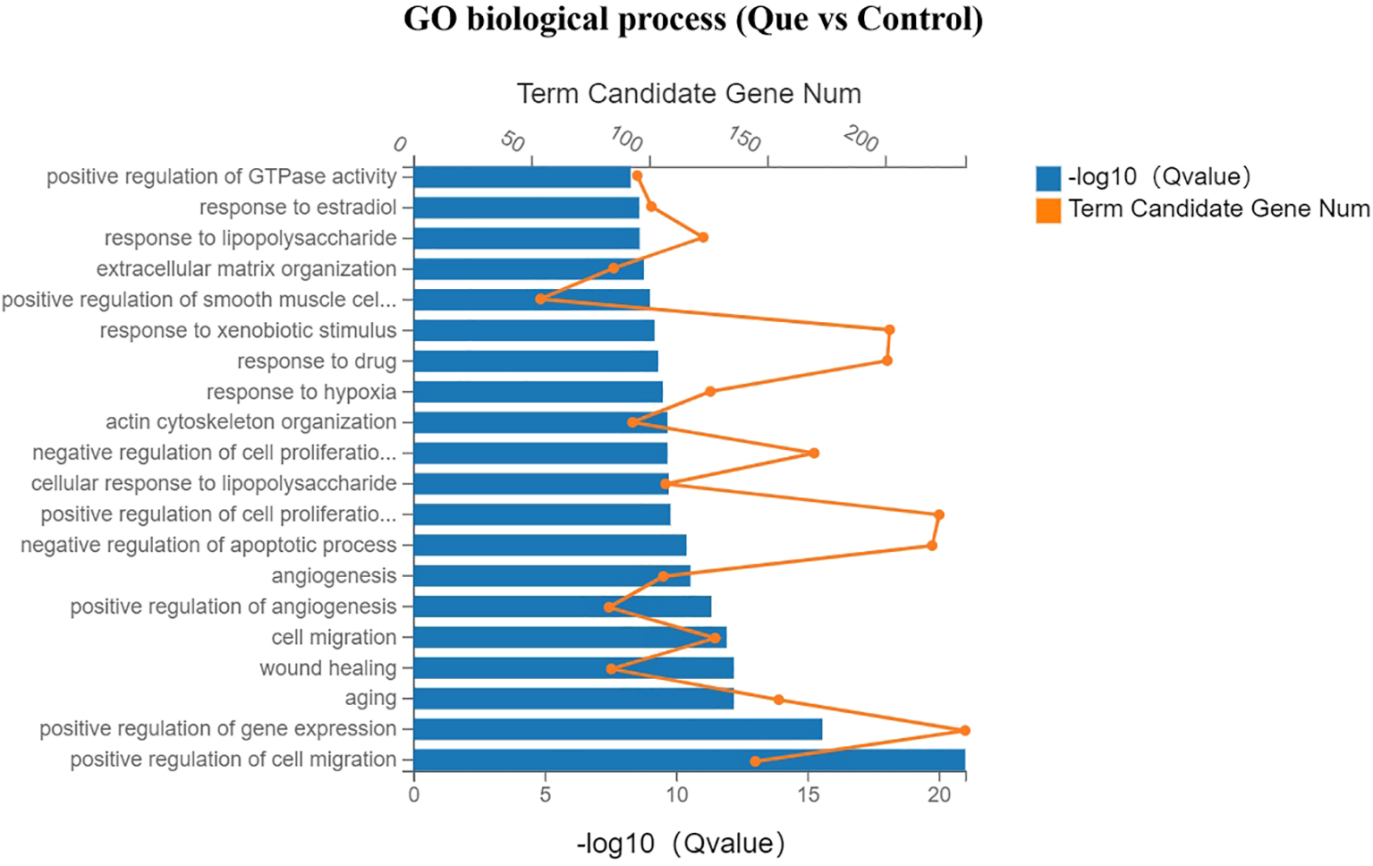
GO enrichment analysis (Cellular Component) for DEGs under direct culture (Que vs Control). Bar chart showing the top enriched GO terms. Yellow dots indicate the number of DEGs annotated to each GO term. Blue bars represent the enrichment significance (-log10 Q value), with longer bars indicating greater statistical significance. Enrichment analysis was performed using the hypergeometric test with FDR correction (Q < 0.05 considered significant).

**Figure 7:**
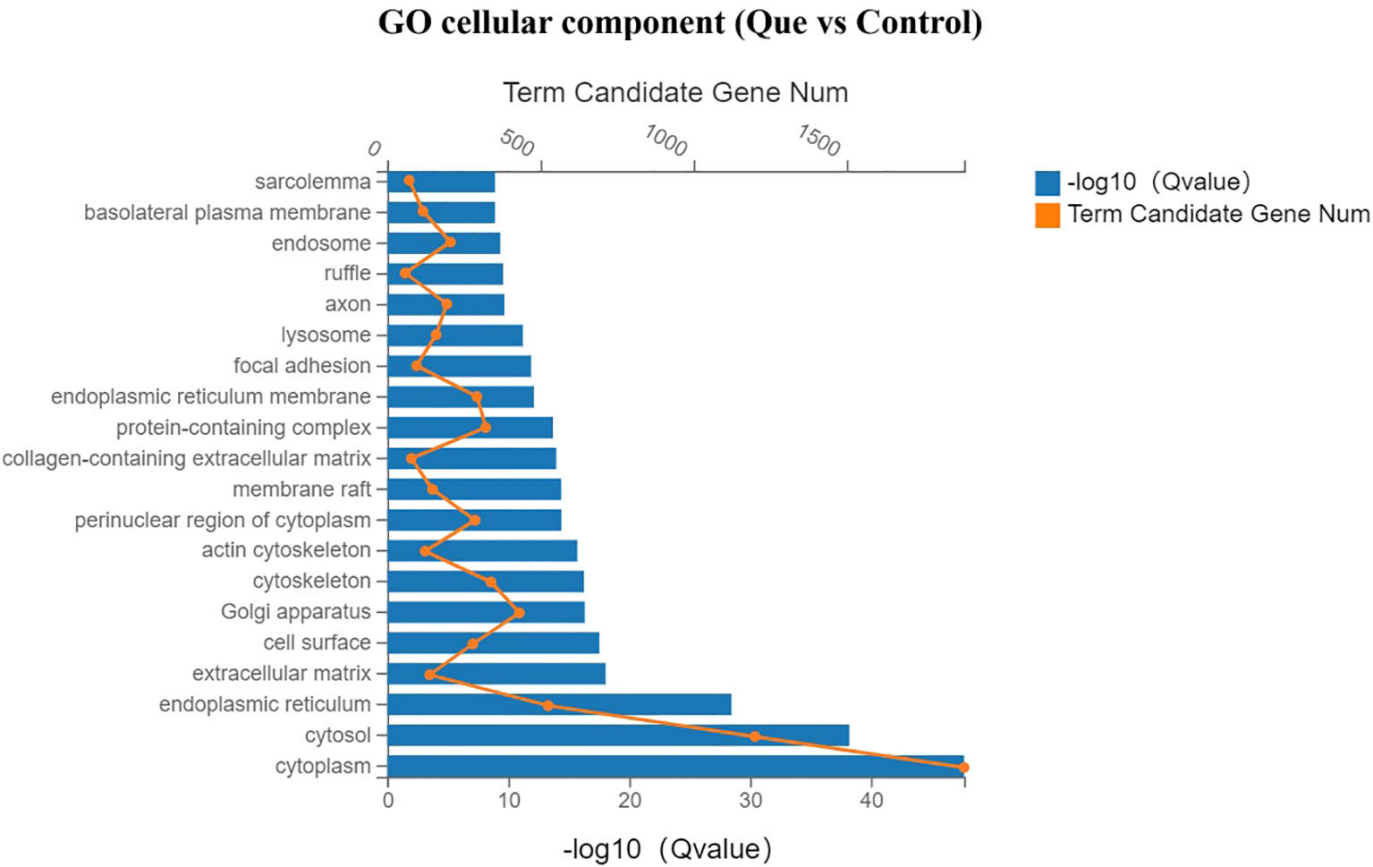
GO enrichment analysis (Biological Process) for DEGs under direct culture (Que vs Control). Bar chart showing the top enriched GO terms. Yellow dots indicate the number of DEGs annotated to each GO term. Blue bars represent the enrichment significance (-log10 Q value), with longer bars indicating greater statistical significance. Enrichment analysis was performed using the hypergeometric test with FDR correction (Q < 0.05 considered significant).

As shown in **Figure 5** (molecular function analysis), Que treatment significantly enriched DEGs associated with protein binding, protein homodimerization activity, integrin binding, actin binding, protein kinase binding, and collagen binding, suggesting enhanced regulation of cellular adhesion and differentiation processes. **Figure 6** (cellular component analysis) revealed that Que predominantly affected genes related to cytoplasm, cytosol, endoplasmic reticulum, Golgi apparatus, extracellular matrix, cell surface, and cytoskeleton, indicating substantial remodeling of intracellular structures. Notably, **Figure 7** (biological process analysis) demonstrated that Que significantly modulated DEGs involved in cell migration, gene expression, angiogenesis, cell proliferation, LPS response, cytoskeleton organization, and extracellular matrix formation—key processes directly linked to osteogenic differentiation.

#### 3.5.3 KEGG pathway enrichment analysis of Que/n-HA/PGCL Nanocomposite Microspheres-Regulated Osteogenic Signaling in BMSCs

**Figure 8:**
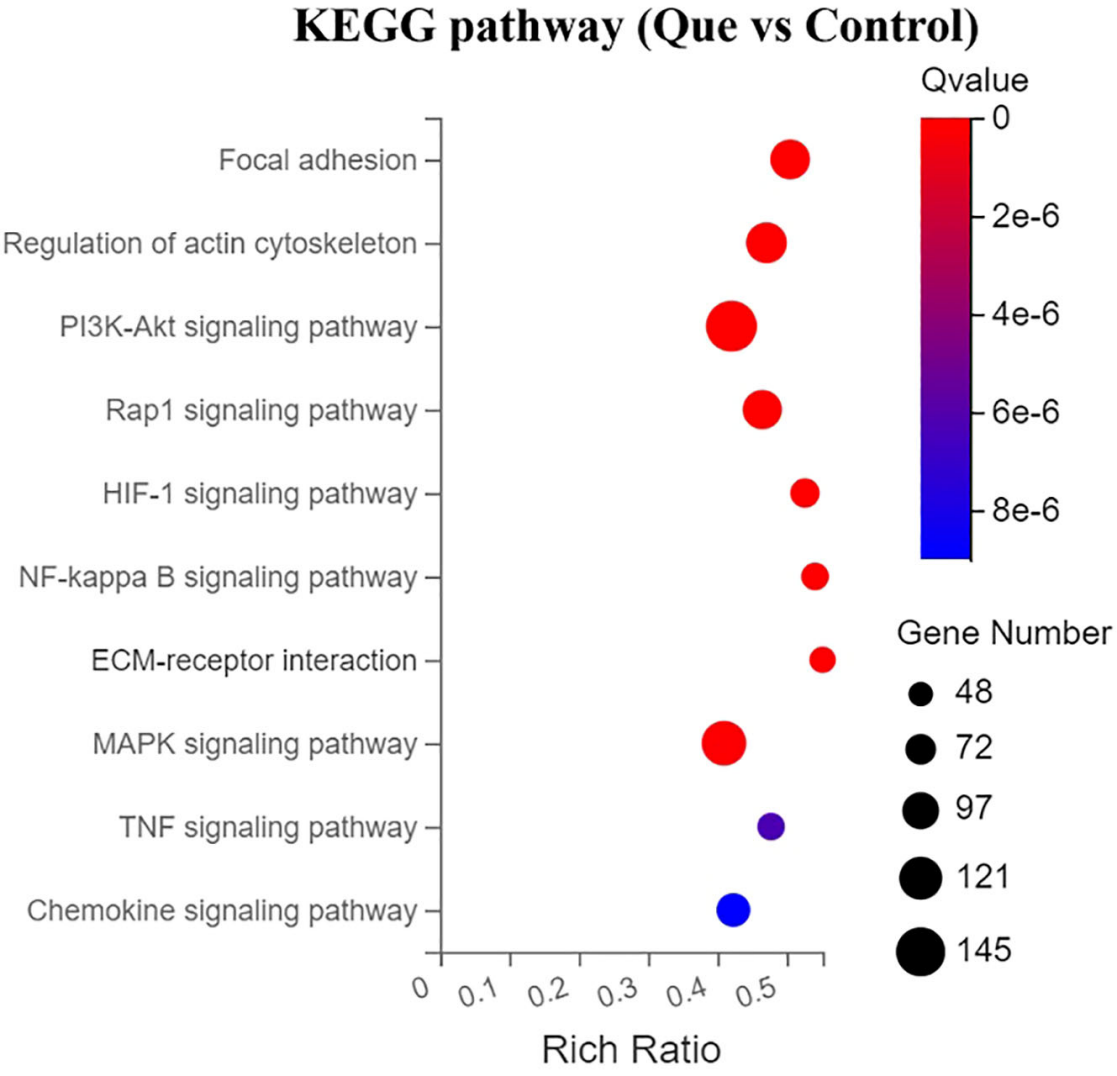
KEGG pathway enrichment analysis for DEGs under direct culture (Que vs Control). Bubble plot of the top 10 enriched signaling pathways. Bubble size represents the number of DEGs involved in each pathway. Bubble color (from blue to red) represents the enrichment significance (-log10 Q value), with red indicating higher significance. X-axis (Rich Ratio) reflects the enrichment degree (number of DEGs in the pathway divided by total genes in the pathway). Enrichment analysis was performed using the hypergeometric test with FDR correction (Q < 0.05).

To further elucidate the molecular mechanisms by which Que/n-HA/PGCL nanocomposite microspheres regulate BMSC osteogenesis, KEGG pathway enrichment analysis was performed on the differentially expressed genes between the Que and Control groups. From the statistically significant results, we selected the top 10 signaling pathways most relevant to osteogenic activity, ranked by their enrichment significance. These pathways were visualized in a bubble chart (**Figure 8**), where bubble size represents the number of differentially expressed genes involved in each pathway, bubble color intensity indicates the enrichment significance (with darker colors representing higher significance), and the horizontal axis (Rich Ratio) reflects the enrichment degree. The analysis revealed that Que/n-HA/PGCL nanocomposite microspheres primarily modulated the following osteogenesis-related signaling pathways: focal adhesion, PI3K-Akt signaling pathway, Rap1 signaling pathway, HIF-1 signaling pathway, ECM-receptor interaction, and MAPK signaling pathway (ranked in descending order of significance). Notably, these pathways are critically involved in cell adhesion, proliferation, survival, and extracellular matrix remodeling—key processes underlying osteogenic differentiation. The strong enrichment of these pathways underscores the multi-faceted regulatory role of Que/n-HA/PGCL nanocomposite microspheres in promoting BMSC osteogenesis through coordinated signaling network modulation.

### 3.6 Regulation of BMSC osteogenic behavior by the immune microenvironment mediated by Que/n-HA/PGCL nanocomposite microspheres

#### 3.6.1 Molecular mechanisms underlying the indirect regulation of BMSC osteogenesis by Que/n-HA/PGCL nanocomposite microspheres via immune modulation

**Figure 9:**
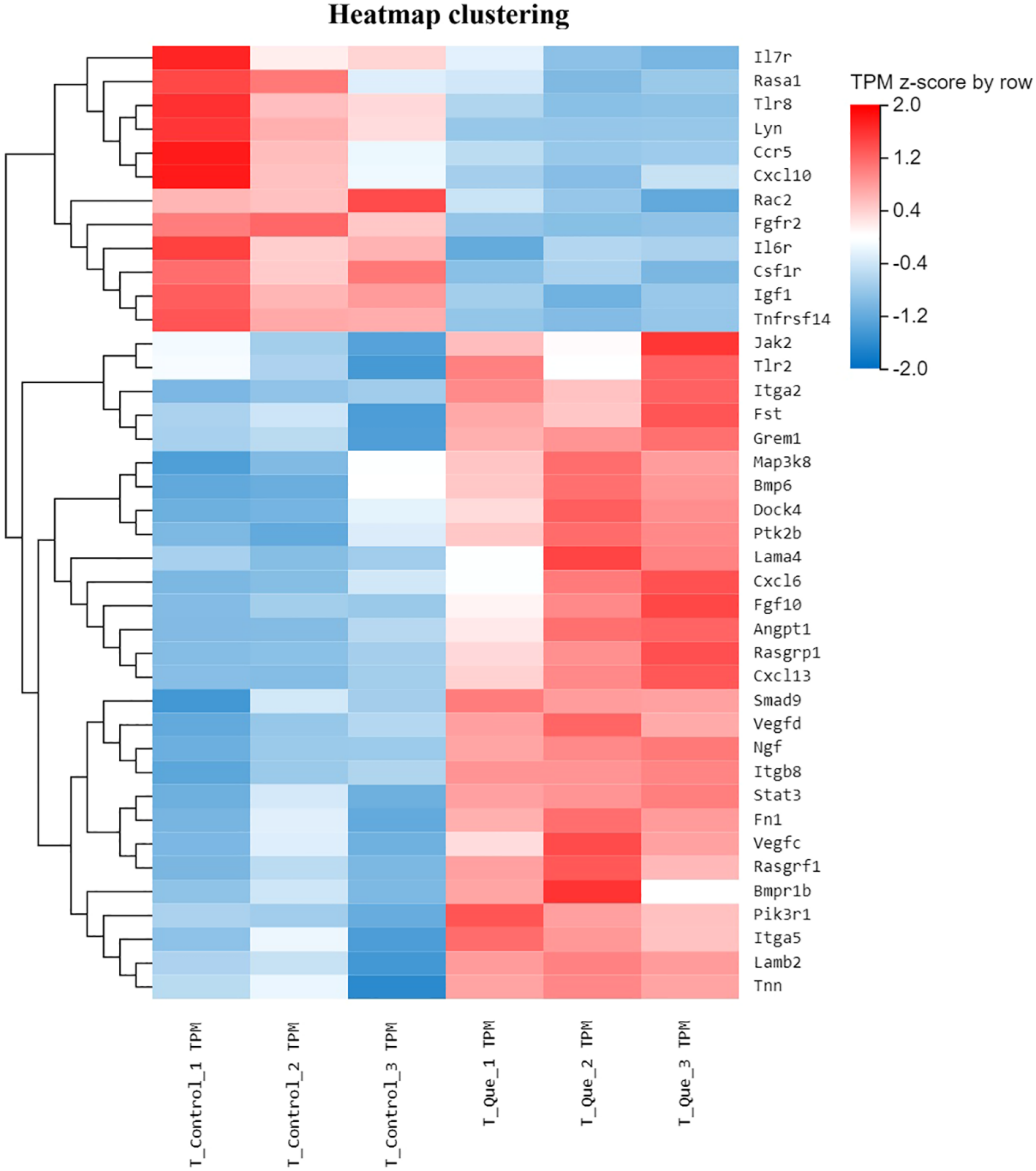
Clustered heatmap of osteogenesis-associated gene expression in BMSCs under immune-mediated conditions (T-Que vs T-Control). Hierarchical clustering of key DEGs associated with osteogenic differentiation under Transwell co-culture conditions. Red indicates upregulated genes; blue indicates downregulated genes. Color intensity represents the degree of differential expression (log2FC scale). Expression values are z-score normalized per gene. Each column represents one biological replicate (n = 3 per group).

As shown in **Figure 9**, in the Transwell co-culture system, Que/n-HA/PGCL nanocomposite microspheres indirectly regulated BMSC osteogenic behavior by modulating RAW264.7 macrophages. Compared with the T-control group, the T-Que group significantly upregulated several osteogenesis-related gene families, including: (1) Integrin family members (Itga2, Itgb8, Itga5); (2) TGF-β superfamily genes and their signaling components (Bmp6, Smad9, Bmpr1b); (3) RAS superfamily gene Rasgrp1; (4) MAPK family member Map3k8; (5) PI3K family member Pik3r1; and (6) Jak family tyrosine kinase Jak2. Additionally, genes associated with cellular processes were upregulated, such as cell division (Dock4), adhesion (Lamb2, Fn1), growth (Ngf, Stat3), and angiogenesis (Angpt1, Vegfc). Conversely, the T-Que group significantly downregulated several inflammation- and osteoclastogenesis-related genes, including inflammatory cytokines and their receptors (Il7r, Il6r, Tnfrsf14), chemokine Cxcl10, and osteoclast regulation factors (Ccr5, Rac2).

**Figure 10:**
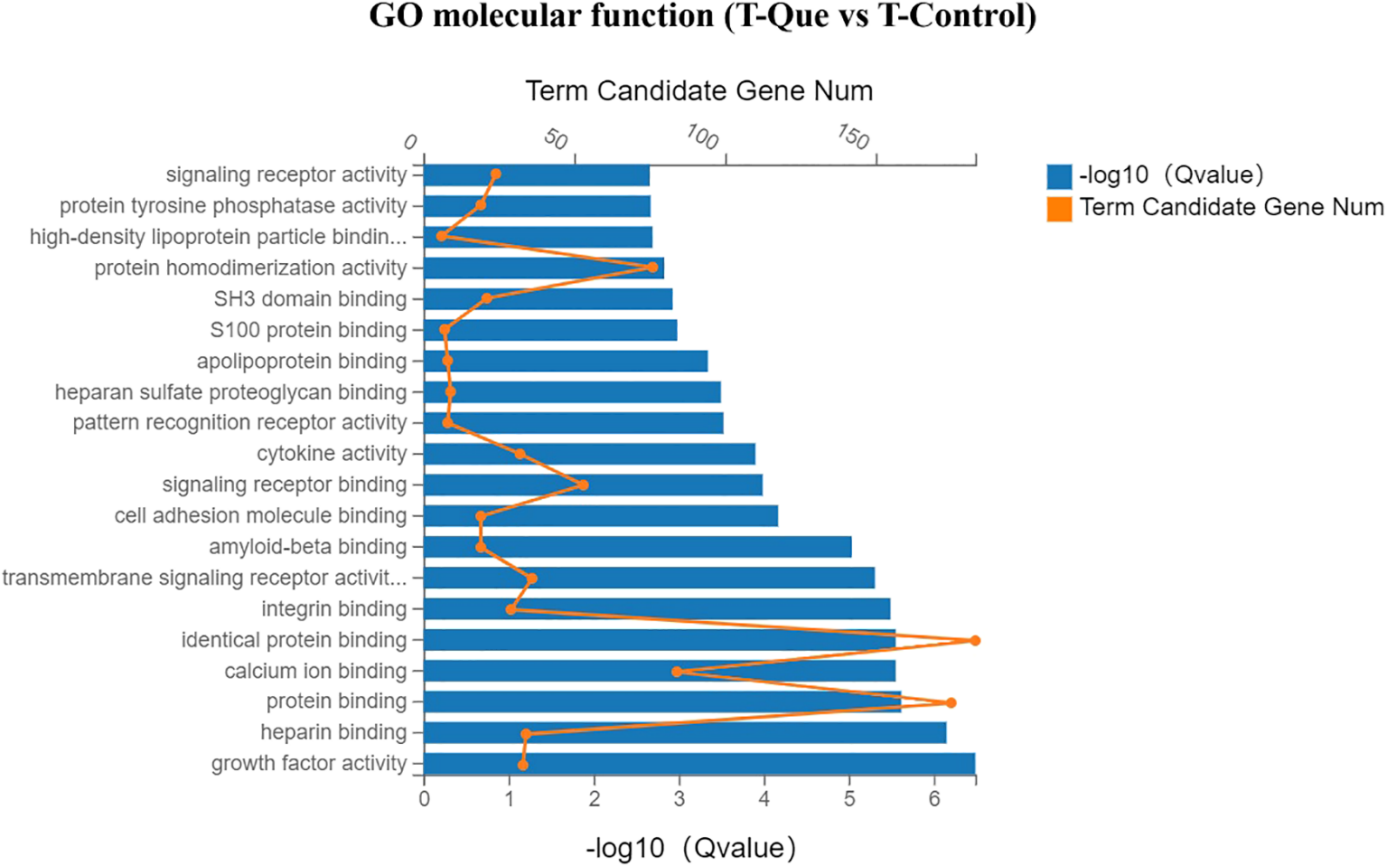
GO enrichment analysis (Molecular Function) for DEGs under immune-mediated conditions (T-Que vs T-Control). Bar chart showing the top enriched GO terms. Yellow dots indicate the number of DEGs annotated to each GO term. Blue bars represent the enrichment significance (-log10 Q value), with longer bars indicating greater statistical significance. Enrichment analysis was performed using the hypergeometric test with FDR correction (Q < 0.05 considered significant).

**Figure 11:**
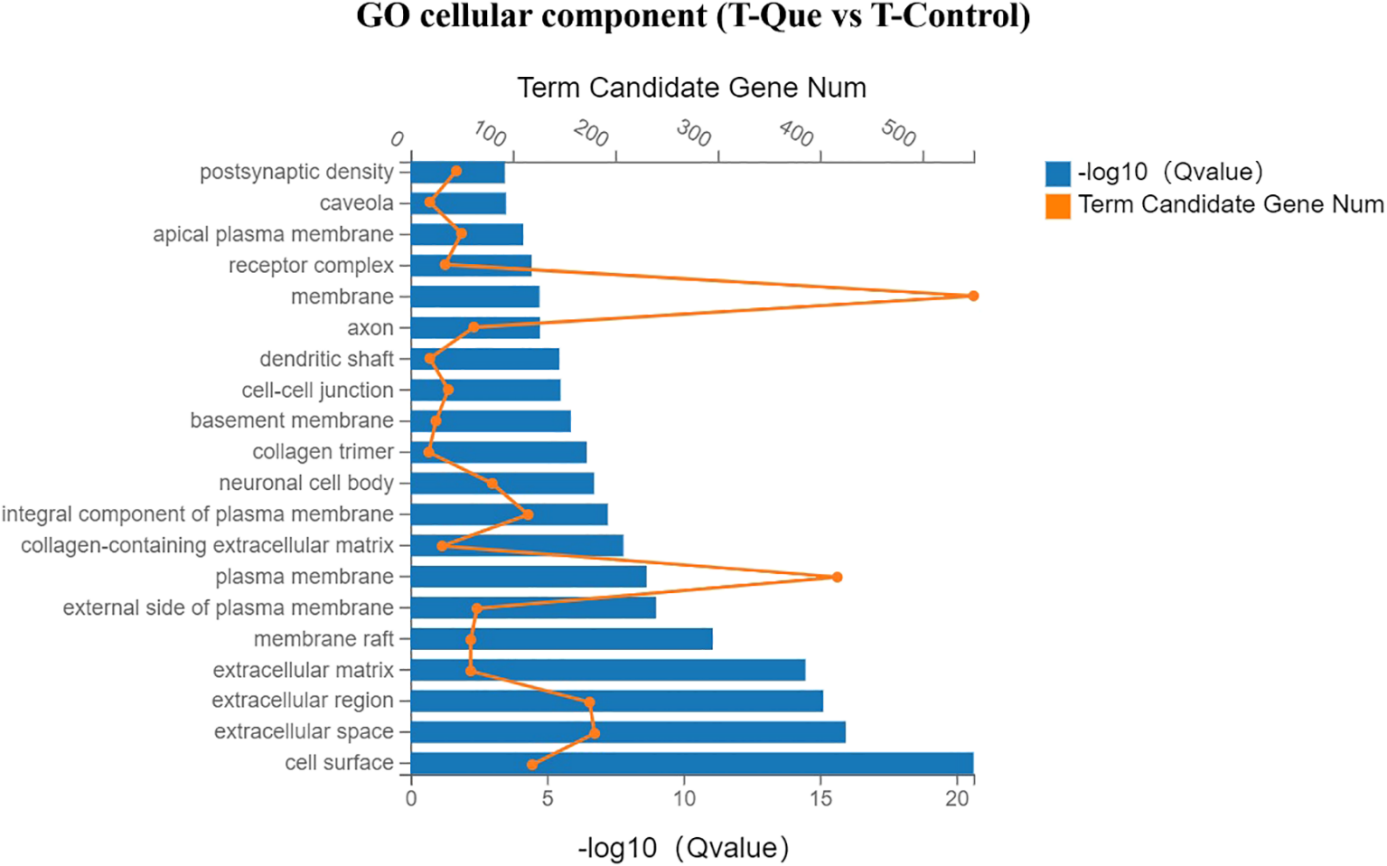
GO enrichment analysis (Cellular Component) for DEGs under immune-mediated conditions (T-Que vs T-Control). Bar chart showing the top enriched GO terms. Yellow dots indicate the number of DEGs annotated to each GO term. Blue bars represent the enrichment significance (-log10 Q value), with longer bars indicating greater statistical significance. Enrichment analysis was performed using the hypergeometric test with FDR correction (Q < 0.05 considered significant).

**Figure 12:**
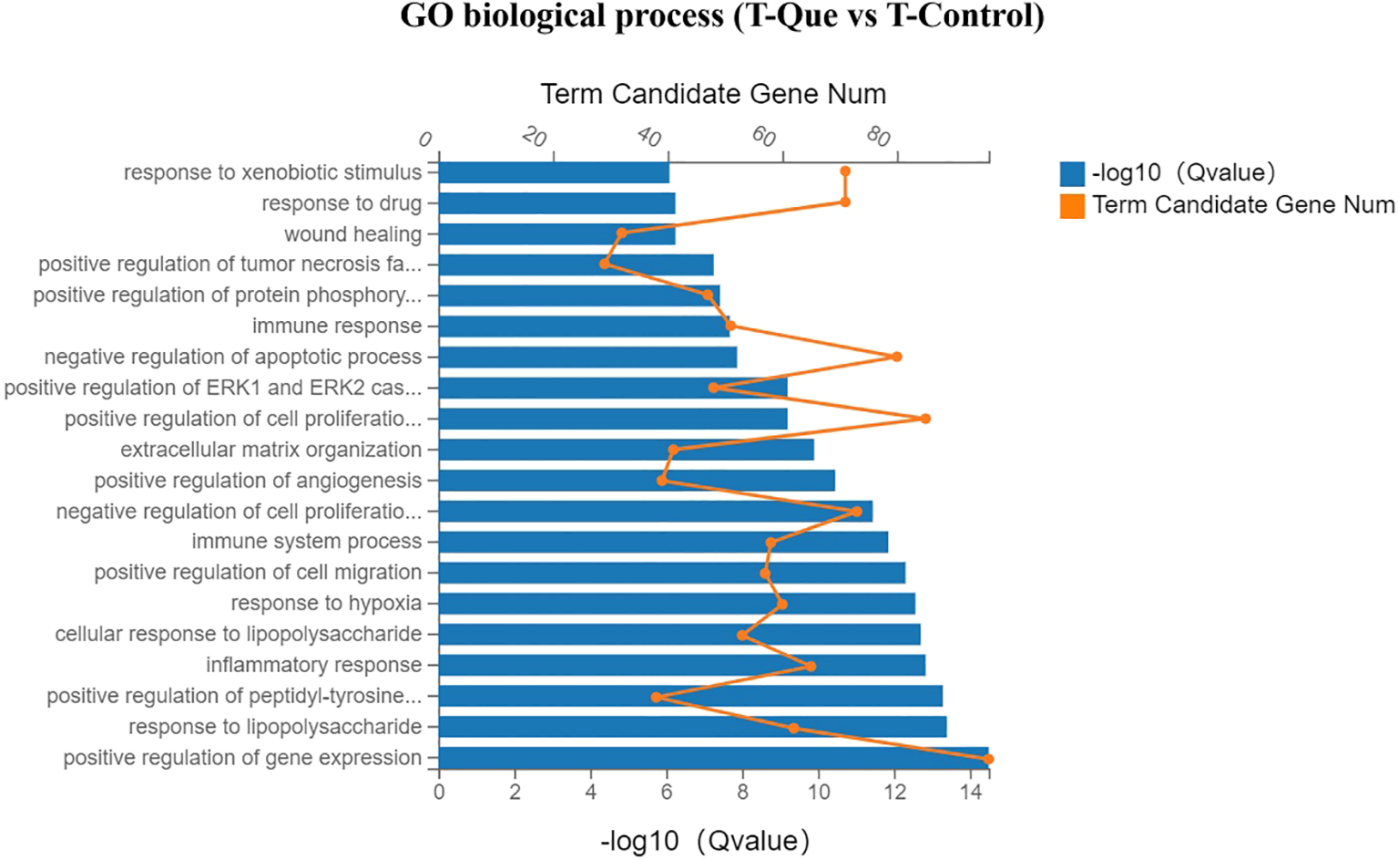
GO enrichment analysis (Biological Process) for DEGs under immune-mediated conditions (T-Que vs T-Control). Bar chart showing the top enriched GO terms. Yellow dots indicate the number of DEGs annotated to each GO term. Blue bars represent the enrichment significance (-log10 Q value), with longer bars indicating greater statistical significance. Enrichment analysis was performed using the hypergeometric test with FDR correction (Q < 0.05 considered significant).

As illustrated in **Figure 10**, the Gene Ontology (GO) enrichment analysis of molecular function revealed that the T-Que group, compared to the T-Control group, predominantly influenced growth factor activity, heparin binding, protein binding, calcium ion binding, protein homodimerization activity, integrin binding, transmembrane signaling receptor activity, and cytokine activity in BMSCs. These findings suggest that T-Que modulates membrane-associated activities mediated by cytokines. As shown in **Figure 11**, the cellular component analysis demonstrated that the T-Que group, relative to the T-Control group, primarily affected genes associated with the cell surface, extracellular region, extracellular matrix, membrane raft, plasma membrane, cell-cell junction, and other membrane-related components in BMSCs, indicating significant alterations in cell surface composition. In the biological process analysis (**Figure 12**), the T-Que group, compared to the T-Control group, mainly regulated gene expression, cellular response to LPS (lipopolysaccharide), peptidyl-tyrosine phosphorylation regulation, inflammatory response, cell migration, cell proliferation, and angiogenesis in BMSCs. These results imply that T-Que exerts an impact on both immune responses and osteogenic differentiation processes.

#### 3.6.3 KEGG pathway enrichment analysis reveals the regulatory effects of Que/n-HA/PGCL nanocomposite microspheres by the immune microenvironment on osteogenic and inflammatory signaling pathways

**Figure 13:**
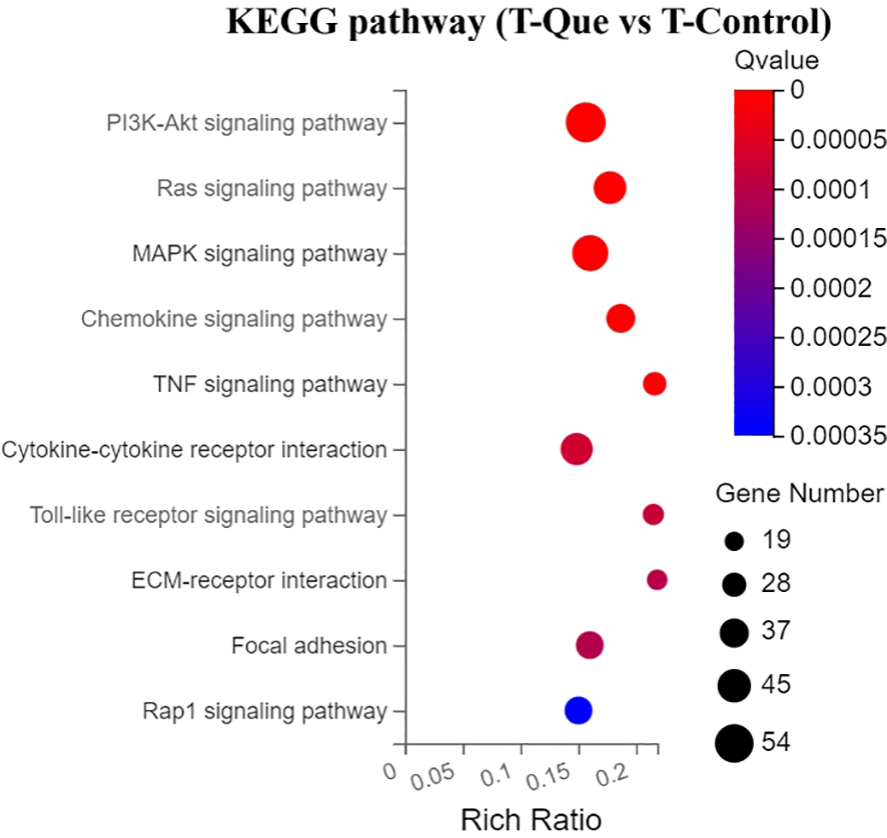
KEGG pathway enrichment analysis for DEGs under immune-mediated conditions (T-Que vs T-Control). Bubble plot of the top 10 enriched signaling pathways. Bubble size represents the number of DEGs involved in each pathway. Bubble color (from blue to red) represents the enrichment significance (-log10 Q value), with red indicating higher significance. X-axis (Rich Ratio) reflects the enrichment degree. Enrichment analysis was performed using the hypergeometric test with FDR correction (Q < 0.05).

To investigate the immunomodulatory effects of Que/n-HA/PGCL nanocomposite microspheres on osteogenic signaling pathways, we performed KEGG pathway enrichment analysis on differentially expressed genes (DEGs) between the T-Que and T-Control groups. From the statistically significant results, we selected the top 10 most significantly enriched pathways related to cellular immunity and osteogenic activity and visualized them in a bubble plot (**Figure 13**). In this plot, the bubble size represents the number of DEGs, the color intensity indicates the significance level, and the Rich Ratio on the x-axis reflects the enrichment degree. In the Transwell co-culture system, Que/n-HA/PGCL nanocomposite microspheres indirectly regulated BMSC behavior by influencing RAW264.7 macrophages. The primary osteogenic-related pathways affected included the PI3K-Akt signaling pathway, Ras signaling pathway, MAPK signaling pathway, ECM-receptor interaction, and TGF-beta signaling pathway, ranked in descending order of significance. Meanwhile, the key inflammatory-related pathways modulated were the Chemokine signaling pathway, TNF signaling pathway, Cytokine-cytokine receptor interaction, and Toll-like receptor signaling pathway. These findings suggest that Que/n-HA/PGCL nanocomposite microspheres exert dual regulatory effects on both osteogenic differentiation and inflammatory responses, potentially contributing to bone regeneration through immunomodulation.

**Figure 14:**
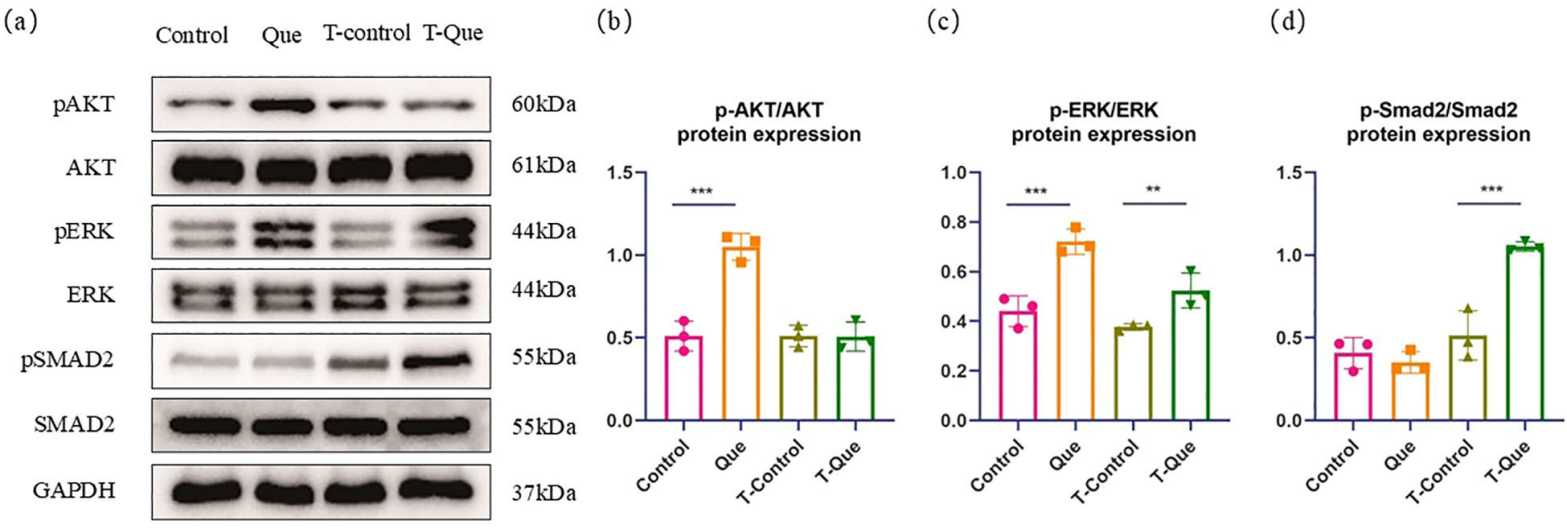
Western blot validation of key signaling pathways regulated by Que/n-HA/PGCL microspheres in BMSC osteogenic differentiation. BMSCs were cultured according to the groupings shown in **Figure 1** for 7 days, after which total protein was extracted for Western blot analysis. **(a)** Representative protein bands of p-Akt, total Akt, p-ERK, total ERK, p-Smad2, and total Smad2 in each group. **(b–d)** Quantitative densitometry analysis of p-Akt/Akt **(b)**, p-ERK/ERK **(c)**, and p-Smad2/Smad2 **(d)**. Data are presented as mean ± SD (n=3). **P < 0.05, ***P < 0.01.

To validate the key signaling pathways identified by RNA-seq at the protein level, we examined the phosphorylation levels of Akt, ERK, and Smad2 in BMSCs using Western blot analysis. As shown in **Figure 14**, under direct culture conditions (BMSCs co-cultured with microspheres), the protein expression levels of p-Akt/Akt and p-ERK/ERK in the Que group were significantly higher than those in the Control group (P < 0.05), confirming that Que/n-HA/PGCL microspheres can directly activate the PI3K-Akt and MAPK signaling pathways in BMSCs.

Under immune-mediated conditions (Transwell co-culture system), the protein expression level of p-ERK/ERK in the T-Que group was significantly higher than that in the T-Control group (P < 0.05), while p-Akt/Akt showed no significant difference between the two groups (P > 0.05). This result indicates that under immune-mediated conditions, the MAPK pathway is activated, but the PI3K-Akt pathway did not show significant changes at the protein level, which may be related to the temporal dynamics of pathway activation or post-transcriptional regulation.

Furthermore, the protein expression level of p-Smad2/Smad2 was significantly higher only in the T-Que group compared to the T-Control group (P < 0.05), while no significant difference was observed between the Que and Control groups under direct culture conditions (P > 0.05). This finding is highly consistent with the RNA-seq results showing enrichment of the TGF-β/Smad pathway exclusively in the immune-mediated group, confirming that Que/n-HA/PGCL microspheres indirectly activate the TGF-β/Smad signaling pathway in BMSCs by modulating macrophage polarization.

In summary, the Western blot results validated at the protein level: (1) activation of the PI3K-Akt and MAPK pathways under direct culture conditions; (2) activation of the MAPK and TGF-β/Smad pathways under immune-mediated conditions. Although RNA-seq suggested enrichment of the PI3K-Akt pathway under immune-mediated conditions, no significant increase in p-Akt was detected at the protein level, suggesting that this pathway may play a more dominant role in the direct osteoinduction phase or may be subject to more complex regulatory mechanisms in the immune-mediated context.

## 4 Discussion

The principal novelty of this study lies in its systematic deconvolution of the osteoimmune mechanisms orchestrated by Que/n-HA/PGCL microspheres. Unlike conventional approaches that assess material effects in mixed cellular systems or focus solely on endpoint phenotypes, our experimental design explicitly separates and analyzes two fundamental biological scenarios: direct BMSC-material contact and macrophage paracrine signaling. This compartmentalization allowed us to generate a ‘signaling atlas’ that distinguishes pathways activated intrinsically in BMSCs (PI3K-Akt and MAPK) from those engaged extrinsically via immune modulation (Ras/MAPK and TGF-β/Smad). This delineation of context-specific pathway engagement represents a significant advancement over the existing literature, providing a mechanistic rationale for how a single biomaterial can perform dual, stage-specific functions during bone healing. It establishes a new paradigm for evaluating and developing osteoimmunomodulatory materials based on their ability to activate precise signaling networks in a cell type- and context-dependent manner.

In this study, a quercetin (Que) sustained-release system was constructed to exert distinct effects during different stages of osteogenesis. The Que/n-HA/PGCL nanocomposite microspheres not only directly regulate the behavior of bone marrow-derived mesenchymal stem/stromal cells (BMSCs), but also indirectly modulate BMSCs behavior by shaping the immune microenvironment through regulation of RAW264.7 macrophages. In this experiment, the optimal ratio of 4 wt% Que/n-HA/PGCL nanocomposite microspheres, as determined in our previous work, was selected as the experimental group, while n-HA/PGCL nanocomposite microspheres served as the control group. Rat BMSCs were used as the experimental model, and high-throughput RNA sequencing (RNA-Seq) was performed after 7 days of co-culture. Differentially expressed genes (DEGs) were screened, followed by Gene Ontology (GO) and Kyoto Encyclopedia of Genes and Genomes (KEGG) pathway enrichment analyses to explore the potential mechanisms by which Que/n-HA/PGCL nanocomposite microspheres regulate osteogenesis. To further validate the key signaling pathways identified by RNA-seq at the protein level, we performed Western blot analysis to examine the phosphorylation levels of Akt, ERK, and Smad2 in BMSCs.

The results showed that all sample data met quality standards, with good biological repeatability and reliability. Compared with the control group, 445 genes were upregulated and 842 genes were downregulated in the Que group. Similarly, when comparing the T-Que group with the T-Control group, 159 genes were upregulated and 273 genes were downregulated. These findings indicate that Que/n-HA/PGCL nanocomposite microspheres exert a certain regulatory effect on BMSCs, both directly and within an immune microenvironment context.

Based on the comprehensive analysis of differentially expressed genes (DEGs) and their enrichment results, the direct regulatory effects of Que/n-HA/PGCL nanocomposite microspheres on osteogenesis can be primarily categorized into the following two aspects:

1. Enhancement of BMSC motility, including cell adhesion, migration, and cytoskeletal remodeling. GO enrichment analysis revealed that, compared with the control group, the Que group significantly influenced intracellular component organization in BMSCs, as well as molecular functions associated with cell adhesion, such as integrin binding and actin-binding. Additionally, biological processes related to BMSC migration, proliferation, cytoskeleton organization, and extracellular matrix (ECM) formation were markedly affected. KEGG pathway analysis provided a more direct visualization of the pathways involved in cell motility regulation. These include the focal adhesion pathway, which regulates cell adhesion and migration; the regulation of actin cytoskeleton pathway, which modulates cytoskeletal dynamics during migration; the ECM-receptor interaction pathway, which is critical for ECM formation; and the Rap1 signaling pathway, which is involved in cell adhesion, proliferation, and chemotaxis. Correspondingly, genes associated with these processes, such as members of the integrin family (Itga7, Itga5, Itga3), adhesion-related genes (Lamc1, Lamb2, Fn1), and the proliferation-related gene Ccnd2, were significantly upregulated.

2. Promotion of osteoblast differentiation. This was evidenced by significant enrichment in GO terms related to molecular functions such as collagen binding and ubiquitin binding, as well as biological processes including angiogenesis, cell proliferation, cytoskeleton organization, and ECM formation. KEGG pathway analysis further highlighted the enrichment of multiple osteogenesis-related signaling pathways, including the PI3K-Akt signaling pathway, Rap1 signaling pathway, HIF-1 signaling pathway, and MAPK signaling pathway. Furthermore, several key genes that facilitate osteogenic differentiation were significantly upregulated, including members of the RAS superfamily (Rasgrp3, Rasgrf1, Rasa2), the mitogen-activated protein kinase (MAPK) family (Map2k1), the hypoxia-inducible factor (HIF) family (Hif1a), proliferation-related genes (Ccnd2), cell growth and angiogenesis-related genes (Stat3, Vegfd), and collagen synthesis-related genes (Col4a3).Conversely, genes associated with processes that are unfavorable for osteogenesis were significantly downregulated. These include members of the matrix metalloproteinase (MMP) family (Mmp14, Mmp3), genes related to NF-κB activation (Nfkb1, Rela), pro-inflammatory cytokines and their receptors such as Il-6, Lif, Il1β, Tnf, and Tnfsf11, as well as chemokines like Ccl4 and Cxcl2.

The immune microenvironment mediated by Que/n-HA/PGCL nanocomposite microspheres plays a regulatory role in osteogenesis, which can be primarily categorized into the following two aspects:

1. Modulation of immune responses to establish a favorable foundation for osteogenesis. GO enrichment analysis revealed that the T-Que group primarily influenced changes in cell membrane components, as well as molecular functions related to transmembrane signaling receptor activity and cytokine activity. It also affected biological processes such as the cellular response to LPS (lipopolysaccharide) and inflammatory responses. KEGG pathway analysis provided a more direct illustration of immune response-related pathways that were significantly impacted, including the chemokine signaling pathway, TNF signaling pathway, cytokine–cytokine receptor interaction, and Toll-like receptor signaling pathway. Furthermore, the T-Que group significantly downregulated the expression of genes encoding inflammatory cytokines and their receptors, such as Il7r, Il6r, and Tnfrsf14.

2. Promotion of osteoblast differentiation. GO analysis showed that the T-Que group mainly affected molecular functions such as protein binding, calcium ion binding, integrin binding, and protein homodimerization activity. It also influenced key biological processes in BMSCs, including gene expression regulation, cell migration, cell proliferation, and angiogenesis. KEGG pathway analysis further highlighted the enrichment of multiple osteogenesis-related signaling pathways, including the PI3K-Akt signaling pathway, Ras signaling pathway, MAPK signaling pathway, and TGF-beta signaling pathway. In addition, the T-Que group significantly upregulated genes associated with osteogenesis, including members of the integrin family (Itga2, Itga5, Itga8), the TGF-beta superfamily and its receptors, as well as downstream signaling molecules such as Bmp6, Smad9, and Bmpr1b. Genes from the RAS superfamily, such as Rasgrp1, and the MAPK family, such as Map3k8, were also upregulated. Furthermore, members of the PI3K family, particularly Pik3r1, and the JAK family of cytoplasmic tyrosine kinases, especially Jak2, were significantly elevated. In addition, genes related to cell division (Dock4), cell adhesion (Lamb2, Fn1), cell growth (Ngf, Stat3), and angiogenesis (Angpt1, Vegfc) were also markedly upregulated in the T-Que group, further supporting the pro-osteogenic effects mediated by the immune microenvironment.

Based on the above findings, the Que/n-HA/PGCL nanocomposite microspheres regulate BMSC behavior both directly and indirectly through modulation of multiple genes and signaling pathways. Bone regeneration is a complex physiological process involving coordinated interactions among various systems, where intricate crosstalk between signaling pathways maintains the delicate balance between osteogenesis and osteoclastogenesis (14). Our results demonstrate that the osteogenic effects of Que/n-HA/PGCL microspheres are primarily mediated through Que release, which enhances BMSC adhesion, migration, proliferation, and differentiation. Mechanistically, the focal adhesion pathway is regulated upstream by Rap1 signaling and ECM-receptor interactions, leading to upregulation of integrin genes (Itga7/5/3) that promote cellular adhesion and migration. Downstream activation of actin cytoskeleton regulation, PI3K-Akt, and MAPK pathways was observed. Western blot results further validated the above findings: Under direct culture conditions, the protein levels of p-Akt/Akt and p-ERK/ERK in the Que group were significantly higher than those in the Control group (P < 0.05), confirming the activation of the PI3K-Akt and MAPK pathways. This finding is consistent with the RNA-seq analysis and previous reports. While previous studies have mainly attributed Que’s effects on BMSCs to MAPK pathway activation (including ERK, p38, and Akt signaling) (15, 16), our findings highlight the crucial role of PI3K-Akt signaling in osteogenic differentiation. Given that PI3K-Akt interacts with MAPK and mTOR pathways (17–20), activation of this pathway may contribute to the observed osteogenic effects. Furthermore, KEGG analysis revealed connections between HIF-1 and VEGF signaling in angiogenesis, suggesting indirect osteogenic promotion (21). The observed inhibition of NF-κB pathway aligns with reported Que-mediated reversal of TNF-α-induced osteogenic impairment (22). Although early ECM interactions (focal adhesion, Rap1, ECM-receptor) significantly influence subsequent cellular activities, these represent general regulators of adhesion/migration rather than osteogenesis-specific pathways. Therefore, we conclude that PI3K-Akt and MAPK pathways likely serve as the primary mechanisms through which Que/n-HA/PGCL microspheres directly regulate BMSC osteogenic differentiation.

In contrast, the osteogenic promotion mediated by the immune microenvironment regulated by Que/n-HA/PGCL nanocomposite microspheres can be attributed to their modulation of BMSC immune responses and osteogenic differentiation. Western blot results provided critical validation for the immune-mediated osteogenic regulation: Under Transwell co-culture conditions, the protein level of p-ERK/ERK in the T-Que group was significantly higher than that in the T-Control group (P < 0.05), confirming the activation of the MAPK pathway. Similarly, the protein level of p-Smad2/Smad2 was significantly elevated in the T-Que group (P < 0.05), which is highly consistent with the RNA-seq finding that the TGF-β/Smad pathway was enriched exclusively in the immune-mediated group, confirming that Que/n-HA/PGCL microspheres indirectly activate the TGF-β/Smad signaling pathway in BMSCs by modulating macrophage polarization. However, it is noteworthy that although RNA-seq suggested enrichment of the PI3K-Akt pathway under immune-mediated conditions, Western blot analysis revealed no significant difference in p-Akt/Akt between the T-Que and T-Control groups (P > 0.05). This discrepancy may be attributed to: (1) Temporal dynamics: PI3K-Akt signaling may be more transient in the immune-mediated context, with its activation peak occurring earlier or later than the 7-day time point examined in this study; (2) Post-transcriptional regulation: Enrichment at the mRNA level does not necessarily translate to activation at the protein level, potentially due to translational inhibition or protein degradation; (3) Pathway crosstalk: The PI3K-Akt pathway enrichment detected by RNA-seq may reflect upstream regulators or crosstalk with other pathways (e.g., Ras-MAPK), rather than direct phosphorylation of Akt itself.

Among the most significantly enriched osteogenic pathways, in addition to the PI3K-Akt and MAPK pathways involved in direct osteogenic regulation, the microspheres also activated the Ras pathway. As an upstream mediator of MAPKs, activated Ras stimulates c-Raf, sequentially phosphorylates MEK1 and ERK1/2, and subsequently triggers downstream substrate phosphorylation, completing the Ras-Raf-MEK-ERK axis (also known as the Ras-MAPK pathway) to regulate cell proliferation and differentiation (23). Analysis of the TGF-β pathway revealed significant upregulation of downstream cascade molecules (e.g., Bmp6, Smad9, Bmpr1b), confirming the critical role of TGF-β/Smads signaling in osteogenesis, consistent with reports that M2 macrophage polarization enhances BMSC osteogenic gene expression via TGF-β/Smads activation (24, 25). Notably, unlike the direct osteogenic regulation, inflammation-related pathways exhibited enhanced significance, emerging as major intracellular/extracellular signaling routes, including chemokine signaling, TNF signaling, cytokine-cytokine receptor interaction, and Toll-like receptor pathways. This aligns with the microspheres’ ability to promote M2 polarization of RAW264.7 macrophages. This transcriptomic profile in BMSCs is consistent with a microenvironment skewed toward an M2-like phenotype, which is known to secrete osteogenesis-favorable cytokines (e.g., IL-10, OSM, VEGF, IGF, TGF-β, BMP2) (25–27), while reducing pro-inflammatory factors detrimental to osteogenesis (e.g., IL-6, TNF-α, IL-1β) from M1 macrophages (28, 29), thereby mitigating inflammatory stimulation and creating a pro-osteogenic milieu. Consistent with our findings, recent studies have shown that biomaterial-induced M2 polarization can involve metabolic reprogramming of macrophages, which in turn enhances the osteogenic differentiation of BMSCs (8). These cytokines engage with the aforementioned inflammatory pathways when acting on BMSCs and indirectly influence osteogenic pathways such as PI3K-Akt, MAPK, and Wnt (30–32). Thus, the Ras-MAPK and TGF-β/Smads pathways likely constitute the key mechanisms through which Que/n-HA/PGCL microspheres indirectly enhance BMSC osteogenic differentiation by remodeling the immune microenvironment, while the role of PI3K-Akt in this context requires further investigation.

Nevertheless, this study has several limitations. First, the RNA seq analysis was conducted *in vitro*, and the transcriptional dynamics *in vivo* may differ due to more complex systemic and local factors. Second, the long term stability, biodegradability, and immunogenicity of the Que/n HA/PGCL microspheres *in vivo* remain to be evaluated. Future work should focus on *in vivo* validation of the identified pathways using animal models of bone defect. Additionally, exploring the synergistic effects of Que/n HA/PGCL microspheres with other osteoinductive agents or growth factors could further enhance bone regeneration efficacy. The integration of single cell RNA seq or spatial transcriptomics in future studies may provide deeper insights into the heterogeneity of cellular responses within the bone healing microenvironment. More importantly, this endeavor is expected to provide novel theoretical foundations for understanding the “immune osteogenic” crosstalk and pave the way for developing next generation smart bone repair materials that integrate immunomodulatory and osteoinductive functions.

## 5 Conclusion

**Figure 15:**
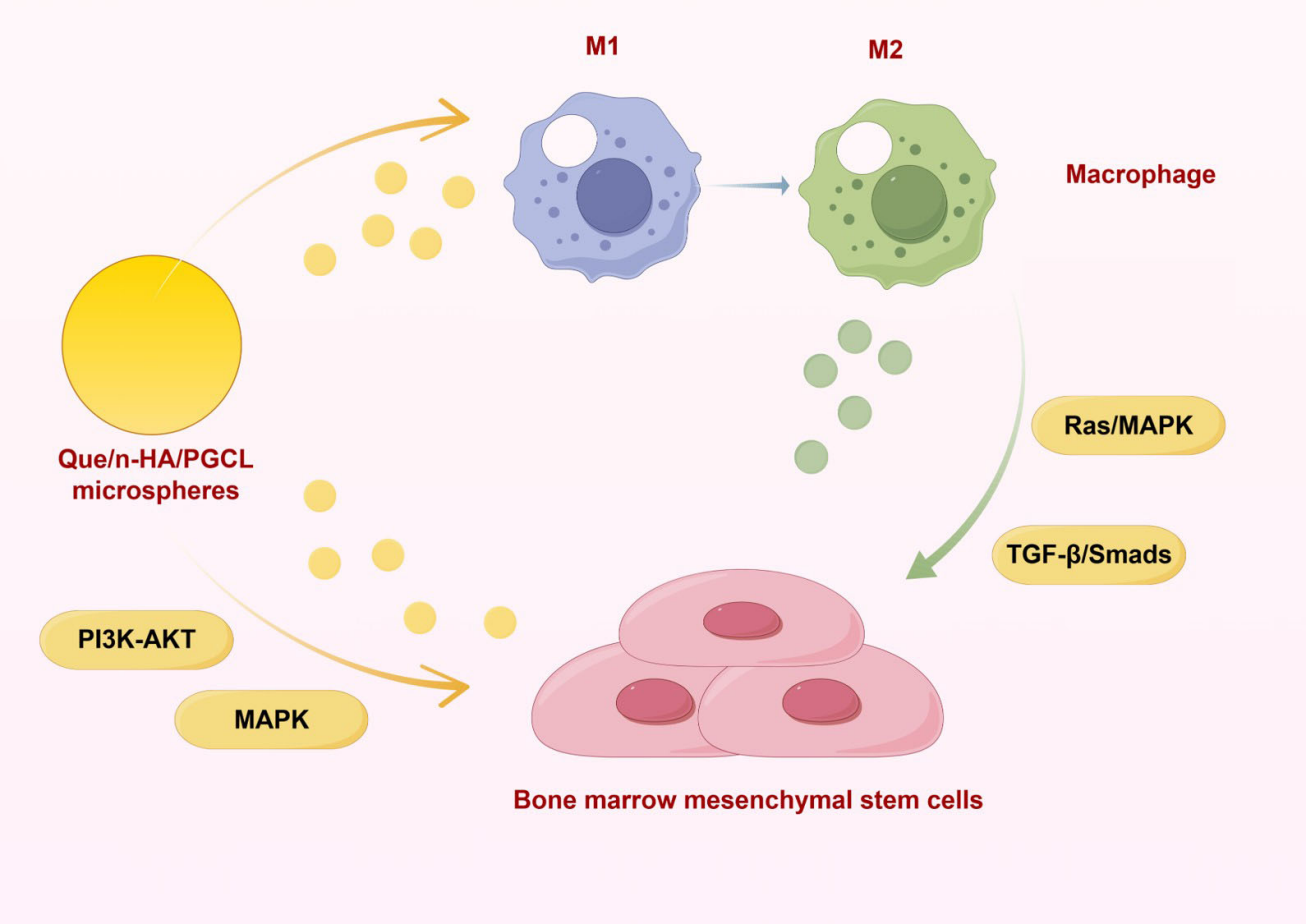
Schematic diagram of the osteogenic regulation mechanism mediated by Que/n-HA/PGCL microspheres. Illustrates the dual-mode action: direct activation of PI3K-Akt/MAPK pathways in BMSCs, and immune-mediated activation of Ras/MAPK, and TGF-β/Smad pathways via M2 macrophage polarization.

In this research, RNA-seq sequencing technology combined with bioinformatics analysis and Western blot validation was employed to preliminarily investigate the effects of Que/n-HA/PGCL nanocomposite microspheres on osteogenesis-related pathways in rat bone marrow-derived mesenchymal stem/stromal cells (BMSCs) under different conditions. As illustrated in **Figure 15**, it was concluded that PI3K-Akt and MAPK pathways are the primary mechanisms for direct regulation, while Ras-MAPK and TGF-β/Smads pathways constitute the key mechanisms through which Que/n-HA/PGCL microspheres indirectly enhance BMSC osteogenic differentiation by remodeling the immune microenvironment.

## Data Availability

The data presented in this study are deposited in the Genome Sequence Archive (GSA) / National Genomics Data Center (CNCB/NGDC), with the BioProject accession number PRJCA050553.
